# Ferroptosis due to Cystathionine γ Lyase/Hydrogen Sulfide Downregulation Under High Hydrostatic Pressure Exacerbates VSMC Dysfunction

**DOI:** 10.3389/fcell.2022.829316

**Published:** 2022-02-03

**Authors:** Ruxi Jin, Ruixue Yang, Changting Cui, Haizeng Zhang, Jun Cai, Bin Geng, Zhenzhen Chen

**Affiliations:** State Key Laboratory of Cardiovascular Disease, National Center for Cardiovascular Diseases, Hypertension Center, Fuwai Hospital, Chinese Academy of Medical Sciences and Peking Union Medical College, Beijing, China

**Keywords:** hydrostatic pressure, ferroptosis, hydrogen sulfide, VSMCs, hypertension

## Abstract

Hydrostatic pressure, stretch, and shear are major biomechanical forces of vessels and play critical roles in genesis and development of hypertension. Our previous work demonstrated that high hydrostatic pressure (HHP) promoted vascular smooth muscle cells (VSMCs) two novel subsets: inflammatory and endothelial function inhibitory VSMCs and then exacerbated VSMC dysfunction. However, the underlying mechanism remains unknown. Here, we first identified that aortic GPX4 (a core regulator of ferroptosis) significantly downregulated association with VSMC novel phenotype elevation in SHR rats and hypertension patients. In primary VSMCs, HHP (200 mmHg) increased iron accumulation, ROS production, and lipid peroxidation compared with normal pressure (100 mmHg). Consistently, the ferroptosis-related gene (*COX-2*, *TFRC*, *ACSL4*, and *NOX-1*) expression was also upregulated. The ferroptosis inhibitor ferrostatin-1 (Fer-1) administration blocked HHP-induced VSMC inflammatory (CXCL2 expression) and endothelial function inhibitory (AKR1C2 expression) phenotyping switch association with elevation in the GPX4 expression, reduction in the reactive oxygen species (ROS), and lipid peroxidation production. In contrast, the ferroptosis inducer RLS3 increased HHP-induced CXCL2 and AKR1C2 expressions. These data indicate HHP-triggering ferroptosis contributes to VSMC inflammatory and endothelial function inhibitory phenotyping switch. In mechanism, HHP reduced the VSMC GSH content and cystathionine gamma-lyase (CSE)/hydrogen sulfide (H_2_S)—an essential system for GSH generation. Supplementation of the H_2_S donor-NaHS increased the VSMC GSH level, alleviated iron deposit, ROS and lipid peroxidation production. NaHS administration rescues both HHP- and RLS3-induced ferroptosis. Collectively, HHP downregulated VSMC CSE/H_2_S triggering GSH level reduction, resulting in ferroptosis, which contributed to the genesis of VSMC inflammation and endothelial function inhibitory phenotypes.

## Introduction

Biomechanical forces within the vasculature contain wall shear stress, circumferential wall tensile stress, and hydrostatic pressure (Takayama et al., 2013), and these biomechanics contribute to pathogenesis and development of hypertension and its complications (Hayashi and Naiki, 2009). Shear stress disorder impaired endothelial function and involved in vascular remodeling, atherosclerosis, plaque progression, and vascular malformations (Zhou et al., 2014). The mechanical cyclic stretch force also contributes to vascular smooth muscle cell functions (e.g., apoptosis, proliferation, and migration), which is crucial in vascular remodeling during hypertension (Cheng et al., 2012; Qi et al., 2018), whereas little attention is paid to hydrostatic pressure regulation. A recent study has shown that high hydrostatic pressure (HHP)–driving VSMCs differentiated into inflammatory subset (elevating CXCL2, CXCL3, and CCL2) and endothelial function inhibitory subset (upregulating AKR1C2, AKR1C3, and PEDF) by single-cell sequencing and exacerbated the VSMC dysfunction (Chen Z. et al., 2021). However, the underlying mechanism remains unknown.

Ferroptosis is a novel type of programmed cell death, characteristic as iron overload, reactive oxygen species (ROS) accumulation, iron-dependent lipid peroxidation, and mitochondrial shrinkage (Wu et al., 2021). Ferroptosis is triggered by the cystine/glutamate antiporter system (Xc^−^ system) dysfunction, glutathione (GSH) depletion, and glutathione-dependent antioxidant enzyme glutathione peroxidase 4 (GPX4) inactivation (Yang and Stockwell, 2016). GSH is an important antioxidant, synthesized from cysteine and oxidizes to oxidized glutathione (GSSG) dependent on GPX4 (Badgley et al., 2020). Meanwhile, GPX4 also reduced cytotoxic lipid peroxide (L-OOH) into nontoxic alcohol L-OH in the presence of the cofactor GSH (Stockwell et al., 2017). Thus, GPX4/GSH is an essential regulatory system in ferroptosis. Recently, many studies highlighted that ferroptosis is a crucial pathophysiological process in chronic diseases such as cancer, diabetes, and nervous system disease (Qiu et al., 2020). Over iron deposits induced ferroptosis causing cardiomyopathy (Gujja et al., 2010) or development of vulnerable plaques (Vinchi et al., 2020). Recombinant human GPX4 (Liu et al., 2021) and ferroptosis inhibitor Fer-1 (Li et al., 2020) treatment attenuated myocardial injuries. These works indicate that ferroptosis contributes to the pathogenesis of cardiovascular diseases.

Cystathionine-γ-lyase (CSE) endogenously produces hydrogen sulfide (H_2_S) exerting a cardiovascular protective role and as the key enzyme for l-cysteine (precursor of GSH) (Kimura, 2021). H_2_S plays an antioxidative role by facilitating the GSH content and removing ROS (Tabassum and Jeong, 2019), also inhibiting GPX4 activity and maintaining the Xc^−^ system stability to mitigate ferroptosis (Chen S. et al., 2021; Wang et al., 2021b). In the present study, we seek to clarify whether VSMC ferroptosis involve into the HHP-induced VSMC phenotyping switch. In mechanism, we investigate the CSE/H_2_S-dependent GSH level in ferroptosis in the HHP condition.

## Materials and Methods

### Human Samples

Human internal mammary arteries were obtained from patients undergoing off-pump coronary artery bypass graft. The internal mammary artery (about 5 mm) was acquired from the patient’s surgical donor artery; then, the paraffin slices were prepared for immunofluorescent staining. This study was approved by Fuwai Hospital Ethics Committee and performed in accordance with ethical standards.

### Animal and Materials

Adult male Wistar–Kyoto (WKY) and spontaneously hypertensive rats (SHR) aged 12–16 weeks were housed in controlled temperature at a 12:12-h light–dark cycle with free access to water and standard diet. All animal protocols complied with all relevant ethical regulations and were approved by the Institutional Animal Care and Use Committee, the Experimental Animal Center, Fuwai Hospital, and the National Center for Cardiovascular Diseases, China. The primary antibodies used in this study are as follows: anti-GPX4 (ab125066, Abcam); anti-CXCL2 (PA5-47015, Invitrogen); anti-AKR1C2 (ab166900, Abcam); anti-CSE (ab151769); ferrostatin-1 (S7243, Selleck); RLS3 (S8155, Selleck); and NaHS (161527, Sigma).

### H_2_S Production Measurement

H_2_S production was measured by the modified methylene blue method, as mentioned previously (Niu et al., 2021). In detail, the inner ring of the Erlenmeyer flask was added with zinc acetate and a filter paper to absorb H_2_S. The outer ring was added with an incubation buffer (potassium phosphate buffer, l-cysteine and pyridoxal 5′-phosphate). The cell lysate in the potassium phosphate buffer was added into the outer ring of the conical flasks. The reaction was performed in 37°C shaking water bath by incubation. After sufficient reaction, trichloroacetic acid was added into the outer ring and incubated for another 1 h to terminate the reaction. Followed by adding N, N-dimethyl-*p*-phenylenediamine sulfate and 10% ammonium ferric sulfate, absorbance at 670 nm was measured by spectrophotometry.

### Cell Culture and Treatment

Primary human aorta vascular smooth muscle cells (HASMCs) were purchased from ScienCell Research Laboratories, Inc. HAMSCs were cultured in a smooth muscle cell medium (1,101, ScienCell Research Laboratories.) containing 2% FBS, 100 U/mL penicillin–streptomycin, and 1% smooth muscle cell growth factor at 37°C in a 5% CO_2_ atmosphere. HASMCs in passages three to six were used in this study. We used a specific hydrostatic pressure device for hydrostatic pressure treatment, as described previously (Chen Z. et al., 2021). HASMCs (∼70% confluence) were set in a hydrostatic pressure device at 100 mmHg or 200 mmHg for culturing for 24 h. For inhibition or activation of ferroptosis, HASMCs were treated with Fer-1 (10 μM, 24 h) or RLS3 (0.1 μM, 6 h) under hydrostatic pressure. For increasing exogenous H_2_S, HASMCs were treated with NaHS (100 μM, 24 h) under hydrostatic pressure.

### Primary Mouse Vascular Smooth Muscle Cell Isolation

Aortic smooth muscle cells were isolated from wild-type male 6–8-week and CSE-knockout mice. Mice were sacrificed by decapitation, followed by separating the aorta *via* removing the fatty tissue and vascular adventitia. Then, the mouse aorta was cut into pieces and allowed for digestion for 8 h in the DMEM containing collagenase type 1. The obtained VSMCs were centrifuged at 300 xg for 5 min, and the pellet was resuspended in the DMEM supplemented with 10% FBS, 2 mM l-glutamine, and 100 U/mL penicillin/streptomycin. The medium was replaced every 2 days. All experimental procedures were conducted in an incubator at a temperature of 37°C, in an atmosphere of 95% air and 5% CO_2_. Primary SMCs with the passage number three to eight were used in the study.

### Iron and GSH Detection

We used two different methods to detect the iron content. After treatment, the intracellular ferrous iron level was assessed with an iron colorimetric assay kit (ab83366, Abcam) according to the manufacturer’s instruction protocol. For FeRhoNox-1 staining, 5 μM of the FeRhoNox-1 staining working solution (MX4558 and MKBIO) was added and incubated in a 37°C, 5% CO_2_ incubator for 60 min after treatment for 24 h in a hydrostatic pressure chamber. After washing three times with PBS, cells were imaged with a confocal microscope. The cellular level of glutathione was determined using the Glutathione Assay Kit (S0035, Beyotime) according to the manufacturer’s instruction protocol.

### Reactive Oxygen Species (ROS) Measurement

ROS was measured by using a fluorometric intracellular ROS kit (MAK143, sigma). HASMCs were incubated with 10 µM DCFH-DA for 1 h. After washing with PBS three times, fluorescence signals were observed by using a fluorescence microscope.

### Mitochondrial Superoxide Measurement

We use the MitoSOX™ Red mitochondrial superoxide indicator (M36008, Invitrogen) to detect ROS in the mitochondria. After treatment, HASMCs were stained with 5 µM MitoSOX Red for 10 min, followed by washing with PBS. The images were observed by using a confocal microscope.

### Lipid Peroxidation Detection

HASMCs were labeled with a lipid peroxidation sensor BODIPY™ 581/591 C11 (D3861, Invitrogen) at 2 µM in a living cell imaging solution for 1 h at 37°C. The images were observed by using a confocal microscope. The fluorescence signals correspond to the oxidized (orange) form of BODIPY 581/591 C11. Oxidation of BODIPY-C11 was calculated by the orange fluorescence intensity.

### Immunofluorescence Staining

For aorta immunofluorescent staining, paraffin sections were incubated with 3% hydrogen peroxide to block the endogenous peroxidase activity. Antigen retrieval was performed for 20 min at 97°C in a citrate buffer, and sections were cooled to room temperature. For HAMSC immunofluorescent staining, cells were fixed with 4% paraformaldehyde for 15 min at room temperature. After washed with PBS three times, cells were permeabilized with 0.5% Triton X-100 for 15 min at room temperature. Subsequently, above tissue sections or cells were blocked with 1% BSA for 1 h and incubated with the primary antibody at 4°C overnight, followed by incubating with a fluorescent-labeled secondary antibody for 1 h at room temperature according to the manufacturer’s instructions. After washing three times with PBS, the nuclei were stained with Hoechst for 8 min. The images of tissues or cells were collected using a fluorescence microscope or by confocal microscopy.

### Western Blot

Cells were lysed in RIPA buffer containing protease- and phosphatase-inhibitor cocktails, followed by sonication and centrifugation. Total protein of cells was quantified by BCA assay. For the assay, 40 μg protein samples were separated by SDS-PAGE and transferred to polyvinylidene fluoride membranes. Then, the PVDF membranes were blocked by 5% defatted milk for 1 h and probed with the primary antibody at 4°C overnight. After washing and incubating with the horseradish peroxidase–conjugated secondary antibody for 1 h at room temperature, the membranes were visualized using the chemiluminescence kit.

### RNA Extraction and qRT-PCR

Total RNA from HASMCs was extracted using TRIzol reagent (Invitrogen) according to the manufacturer’s instructions. cDNA was reverse transcribed from 1 µg total RNA by using the cDNA synthesis kit (K1622, Thermo scientific). The real-time PCR was performed in a final volume of 20 μl, which contained 10 μl of the 2×SYBR Mix (Yeasen, China), 1 μl of forward and reverse primers, respectively, 4 μl of template cDNA, and 5 μl of RNase-free H_2_O. A sample without cDNA was subjected to an identical protocol as a negative control. The PCR amplification was accomplished with initial denaturation at 95°C for 10 min, followed by 40 cycles at 95°C for 15 s and 1 min at 60°C for primer annealing and extension. The relative expression of target genes was normalized to that of GAPDH and analyzed by the 2^−ΔΔCT^ method. The primer sequences used for qRT-PCR are provided in Supplementary Table S1.

### Statistical Analysis

All data are presented as the mean ± SD. The statistical significance of differences between groups was analyzed by the *t*-test or by one-way analysis of variance (ANOVA) when more than two groups were compared. *p* values <0.05 were considered as statistically significant.

## Results

### Elevation of Ferroptosis in Aortic Media of SHR and Hypertensive Patients Associated With VSMC Novel Phenotypes

**FIGURE 1:**
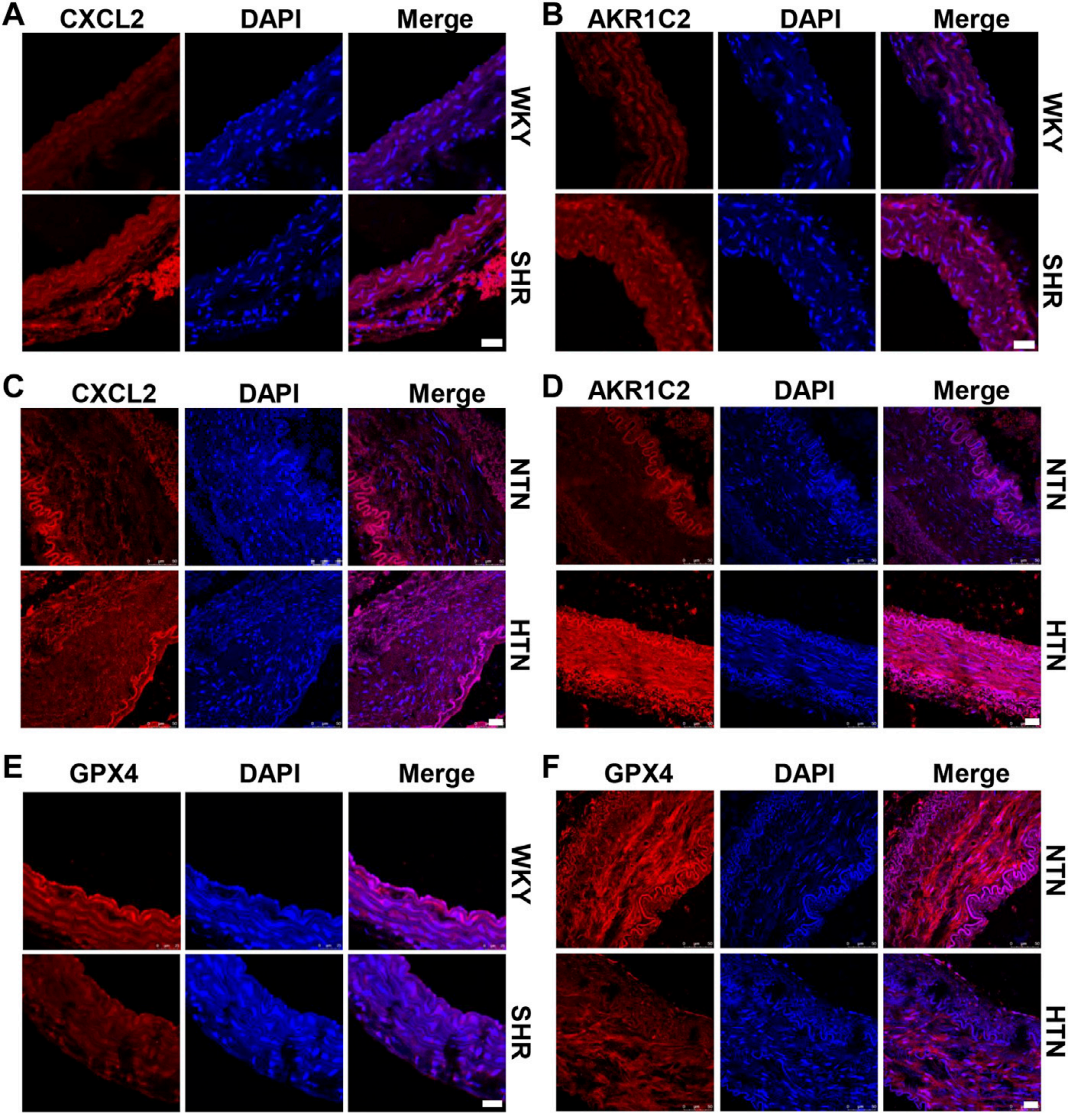
GPX4 expression positively correlated with the VSMC-specific phenotype in SHR and hypertensive patients. Immunofluorescent staining of CXCL2 **(A)** and *AKR1C2* **(B)** in the arterial media of SHR. Immunofluorescence of CXCL2 **(C)** and *AKR1C2* **(D)** on paraffin-embedded human normotension (NTN) and hypertension (HTN) internal mammary arteries. The GPX4 expression was detected by immunofluorescent staining in SHR **(E)** and human hypertensive patients **(F)**. Nuclei were counterstained with Hoechst. Scale bar = 25 µM.

High hydrostatic pressure drives VSMC differentiation into two novel phenotypes: the inflammatory phenotype (marker genes are CXCL2/CXCL3/CCL2) and endothelial function inhibitory phenotype (marker genes are AKR1C2/AKR1C3/PEDF), and promote VSMC dysfunction (Chen Z. et al., 2021). Compared with WKY rats, SHR exhibits CXCL2 and AKR1C2 increase in the media of aorta (Figures 1A,B and Supplementary Figure S1A). Similarly, CXCL2 and AKR1C2 expressions were significantly enhanced in the human internal mammary artery of hypertensive patients in comparison with normotensive patients (Figures 1C,D and Supplementary Figure S1B). To investigate ferroptosis involved in HHP-induced VSMC novel phenotypes, we measured the GPX4 (a key enzyme of ferroptosis) protein level. Here, we showed that aortic media GPX4 dramatically downregulated in the SHR and hypertensive patients compared with the related control (Figures 1E,F and Supplementary Figure S1A–B). These results indicated that ferroptosis associated with the VSMC phenotype switch in the hypertensive animal model and hypertensive patients.

### High Hydrostatic Pressure Induces Ferroptosis

**FIGURE 2:**
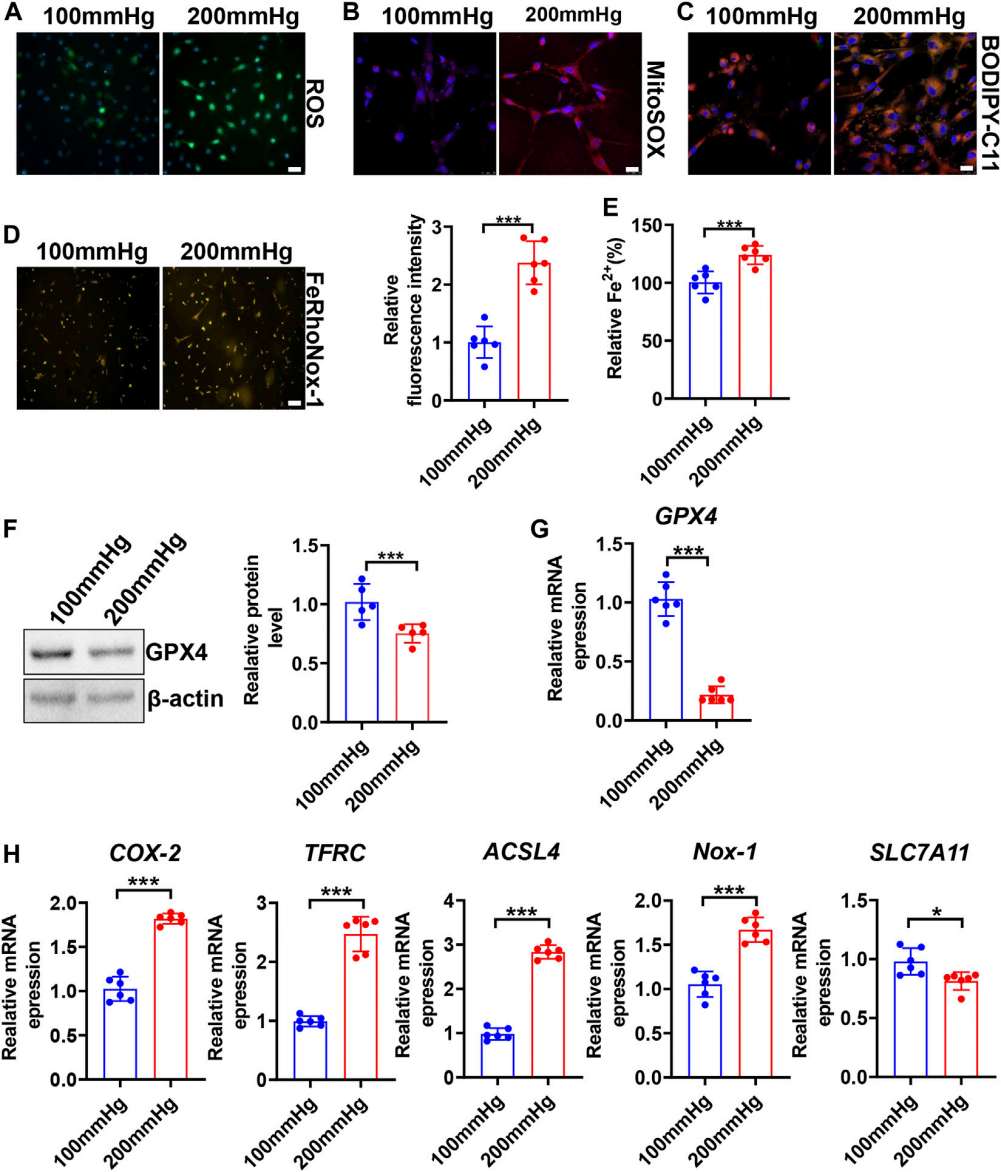
High hydrostatic pressure promoted ferroptosis. HASMCs were treated with hydrostatic pressure for 24 h. Immunofluorescent staining of total ROS, scale bar = 50 µM **(A)**. MitoSOX Red staining of HASMCs for mitochondrial ROS, scale bar = 25 µM **(B)**. BODIPY-C11 staining measured the lipid peroxidation; orange indicated oxidized C11, scale bar = 25 µM **(C)**. Iron level was detected by FeRhoNox-1 staining **(D)** and iron assay kit **(E)**, scale bar = 100 µM. Western blot assay measured the GPX4 protein level; the left panel is the representative image, and the right panel is the statistical graph **(F)**. The mRNA levels of GPX4 **(G)**, COX-2, TFRC, ACSL4, NOX-1, and SLC7A11 were measured by real-time PCR **(H)** under HHP conditions. Nuclei were counterstained with Hoechst. *p < 0.05, ***p < 0.001.

To seek whether HHP induces VSMC ferroptosis, we constructed a specific cell culture chamber and set 200 mmHg as HHP and 100 mmHg as normal (simulates hydrostatic pressure of the aortic wall in normotensive and hypertensive individuals). For evaluation ferroptosis, HHP (200 mmHg)-treatment increased the VSMC total ROS production (Figure 2A and Supplementary Figure S2A), mitochondria-derived ROS releasing by MitoSOX Red staining (Figure 2B and Supplementary Figure S2B), lipid peroxidation production by BODIPY 588/591 C11 (Figure 2C and Supplementary Figure S2C), iron accumulation by FeRhoNox-1 staining (Figure 2D), and iron content by the iron assay kit (Figure 2E) in comparison to normal pressure. In line with the ROS enhancing and lipid peroxidation, the GPX4 protein (Figure 2F) and mRNA level (Figure 2G) also lowered under the HHP condition. Indeed, the ferroptosis-related gene (*COX-2*, *TFRC*, *ACSL4*, and *NOX-1*) mRNA expression was upregulated, and SLC7A11 mRNA was downregulated while exposed to HHP (Figure 2H). Overall, these results indicate high hydrostatic pressure promotes VSMC ferroptosis.

### Ferroptosis Contributes to the HHP-Induced Specific VSMC Phenotype Switch

**FIGURE 3:**
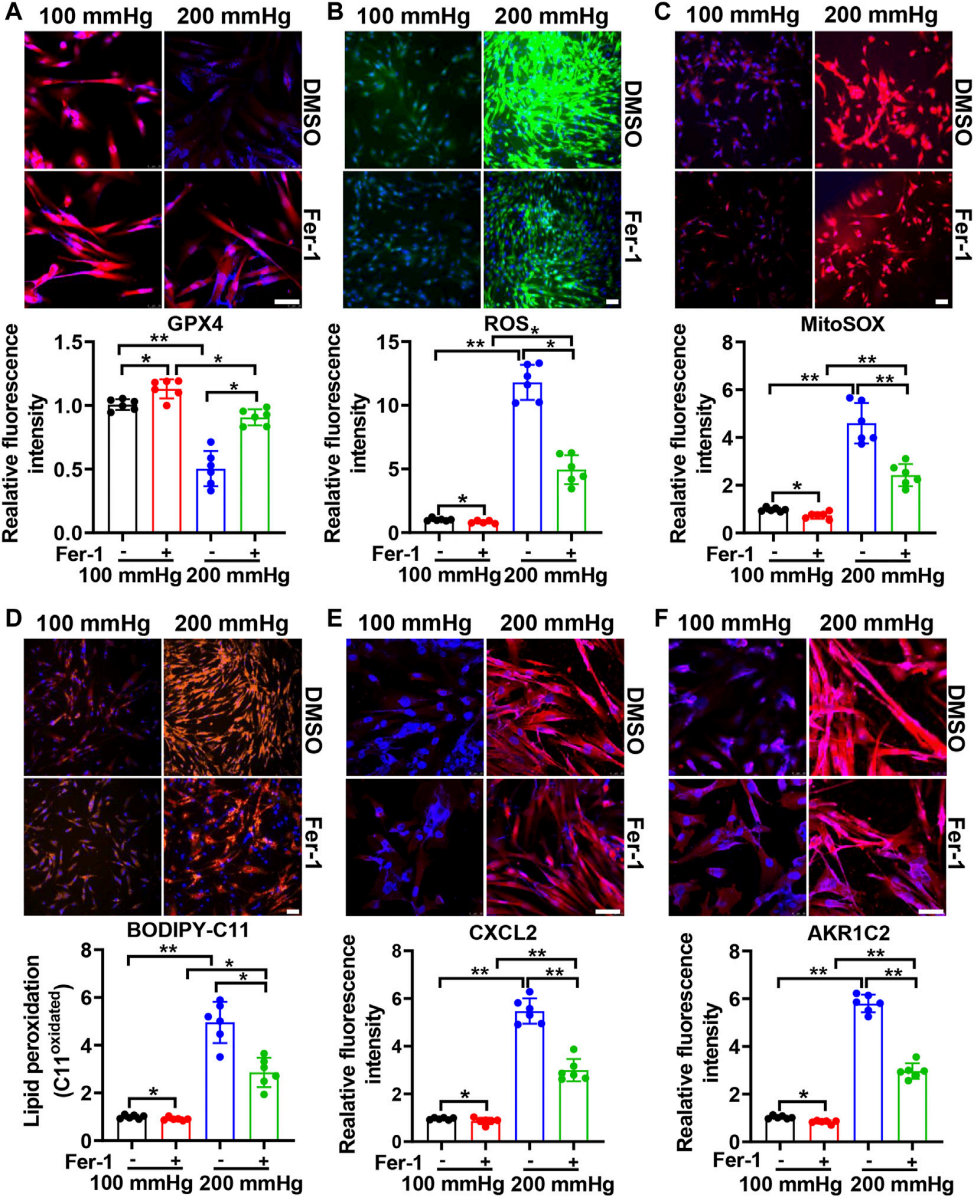
Ferroptosis inhibitor Fer-1 mitigates the HHP-induced VSMC phenotype switch. HASMCs in a 100 mmHg or 200 mmHg incubator were treated with or without 10 μM Fer-1 for 24 h. The GPX4 protein level was evaluated by immunofluorescent staining; the upper panel is the representative image, and the lower panel is the statistical graph **(A)**. ROS production was determined by the ROS staining kit; the upper panel is the representative image, and the lower panel is the statistical graph **(B)**. Mitochondrial ROS was visualized by use of the fluorescent probe MitoSOX Red **(C)**. BODIPY 581/591 C11 lipid oxidation in HASMCs was measured by immunofluorescent staining; the statistical graph is the oxidized (orange signal) BODIPY 581/591 C11 fluorescence intensity **(D)**. Confocal images of the CXCL2 expression **(E)** and AKR1C2 expression **(F)** in HASMCs. Nuclei were counterstained with Hoechst. All scale bar = 50 μM *p < 0.05, **p < 0.01, ***p < 0.001.

To investigate the effect of ferroptosis on HHP-induced specific VSMC subsets, we treated HASMCs with the ferroptosis suppressor Fer-1. Fer-1 supplementation upregulated the VSMC GPX4 expression under normal or high hydrostatic pressure conditions (Figure 3A). In line with GPX4 upregulation, Fer-1 administration also blocked HHP-stimulated ROS production (Figure 3B), mitochondrial ROS production (Figure 3C), and lipid oxidation (Figure 3D), similar to other ferroptosis inducers (Dixon et al., 2012). These results confirmed Fer-1 blocked HHP-associated ferroptosis. In association with ferroptosis inhibition, Fer-1 also blocked HHP-induced VSMC CXCL2 (Figure 3E) and AKR1C2 (Figure 3F) elevation.

**FIGURE 4:**
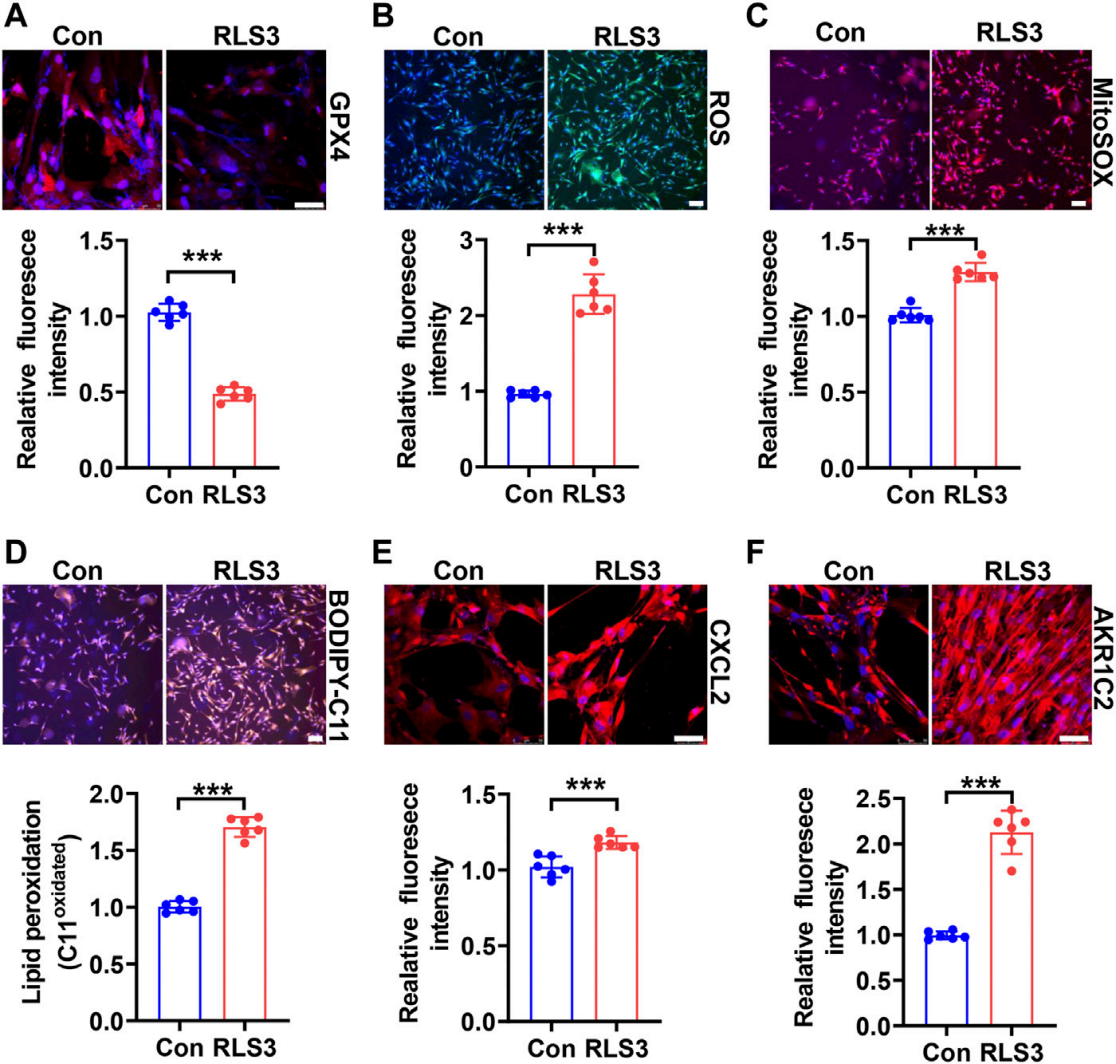
Ferroptosis inducer RLS3 aggravates VSMC dysfunction in response to HHP. HASMCs were treated with RLS3 0.1 μM for 6 h in the presence of 200 mmHg. Immunofluorescent staining of GPX4 (red) in HASMCs **(A)**. Intracellular ROS were measured by ROS staining; the upper panel is the representative image, and the lower panel is the statistical graph **(B)**. HASMCs were labeled with the MitoSOX Red probe to detect mitochondrial ROS **(C)**. Confocal images of the BODIPY 588/591 C11 staining; lipid oxidation was measured by the relative intensity of oxidized (orange fluorescence signal) BODIPY 581/591 C11 **(D)**. CXCL2 **(E)**, and AKR1C2 **(F)** protein levels were detected by immunofluorescent staining. All scale bar = 50 μM ***p < 0.001.

Since HHP-induced ferroptosis is in part of the GPX4/GSH pathway, we used a ferroptosis inducer RLS3 (GPX4 inactivation) to confirm its role in the VSMC novel phenotype switch. Under 200 mmHg hydrostatic pressure condition, RLS3 ulteriorly reduced the GPX4 expression (Figure 4A). Consistently, RLS3 exacerbated ferroptosis by increasing ROS production (Figure 4B), mitochondrial ROS production(Figure 4C), and lipid oxidation (Figure 4D). Correspondingly, RLS3 further increases the HHP-induced VSMC CXCL2 (Figure 4E) and AKR1C2 expression (Figure 4F). Taken together, these data indicated that ferroptosis contributed to the pathophysiological process of HHP-induced VSMC-specific phenotypes.

### CSE/H_2_S Alleviate HHP-Induced VSMC Ferroptosis

**FIGURE 5:**
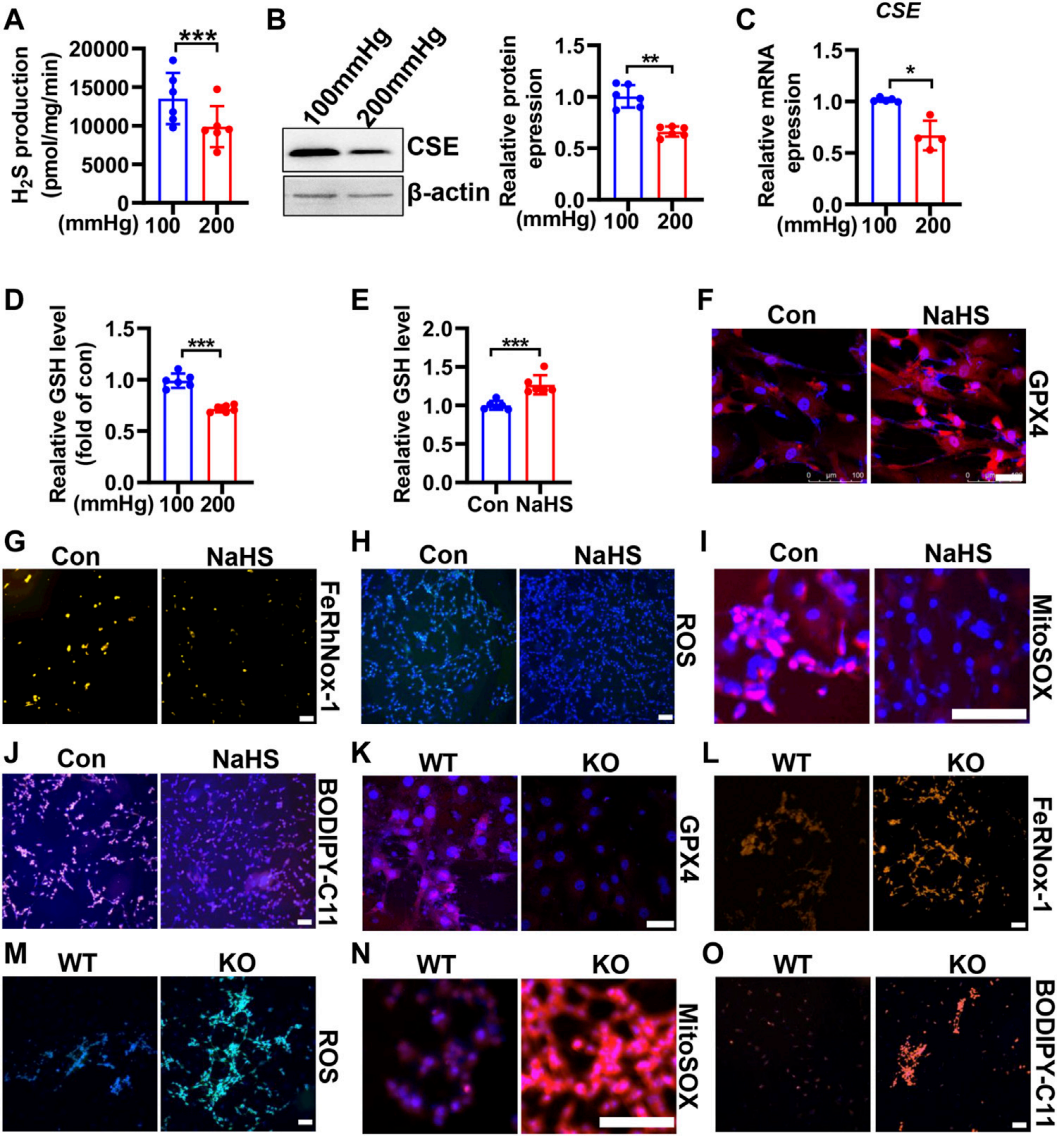
CSE/H_2_S attenuates HHP-induced ferroptosis in VSMCs. H_2_S production was measured by the modified methylene blue method under 100 or 200 mmHg **(A)**. Western blot assay analysis of the CSE expression under hydrostatic pressure; the left panel is the representative image, and the right panel is the statistical graph **(B)**. CSE mRNA level under hydrostatic pressure **(C)**. GSH assay kit measured the GSH level in response to hydrostatic pressure **(D)**. After NaHS administration (0.1mM, 24 h) in the presence of 200 mmHg, the GSH content was measured **(E)**. Representative image of GPX4 staining after NaHS treatment under HHP conditions **(F)**. Effect of NaHS on Fe^2+^ **(G)**, ROS **(H)**, mitochondrial ROS **(I)**, and lipid oxidation **(J)** under 200 mmHg by immunofluorescent staining. In isolated primary mouse VSMCs, the effect of deletion of CSE on the GPX4 expression **(K)**, iron level **(L)**, ROS production **(M)**, mitochondrial ROS production **(N)**, and lipid peroxidation **(O)** under HHP conditions by immunofluorescent staining. All scale bar = 50 μM *p < 0.05, **p < 0.01, ***p < 0.001.

CSE/H_2_S plays a crucial role in GSH synthesis due to controlling the GSH precursor-l-cysteine generation, and CSE/H_2_S plays an essential protection in VSMC function (Kimura, 2021). To investigate whether CSE/H_2_S involved in the modulation of HHP-induced ferroptosis, we first measured the CSE/H_2_S changes exposed to HHP (200 mmHg). As Figure 5A showed, HHP treatment for 48 h dramatically reduced the HASMC H_2_S production (about 27%). The CSE protein (Figure 5B) and mRNA expression (Figure 5C) were also downregulated under HHP. The association with CSE/H_2_S downregulation and the intracellular GSH level reduced about 28% (Figure 5D).

In contrast, supplementation H_2_S donor-NaHS under HHP culture, upregulated the GSH level and GPX4 expression (Figures 5E,F and Supplementary Figure S3A) but lowered the HASMC iron concentration (Figure 5G and Supplementary Figure S3B), ROS production (Figure 5H and Supplementary Figure S3C), mitochondrial ROS production (Figure 5I and Supplementary Figure S3D), and lipid peroxidative production (Figure 5J and Supplementary Figure S3E). Next, we isolated primary CSE-knockout mouse VSMCs to confirm its role in HHP-induced ferroptosis. Under HHP conditions, CSE deficiency further decreased the GPX4 expression (Figure 5K and Supplementary Figure S3F), enhanced iron accumulation (Figure 5L and Supplementary Figure S3G), cellular and mitochondrial ROS (Figure 5M–N and Supplementary Figure S3H–I), and lipid peroxidative production (Figure 5O and Supplementary Figure S3J). Our present data highlight HHP decreases CSE/H_2_S causing GSH level reduction attribution to pathogenesis of VSMC ferroptosis.

**FIGURE 6:**
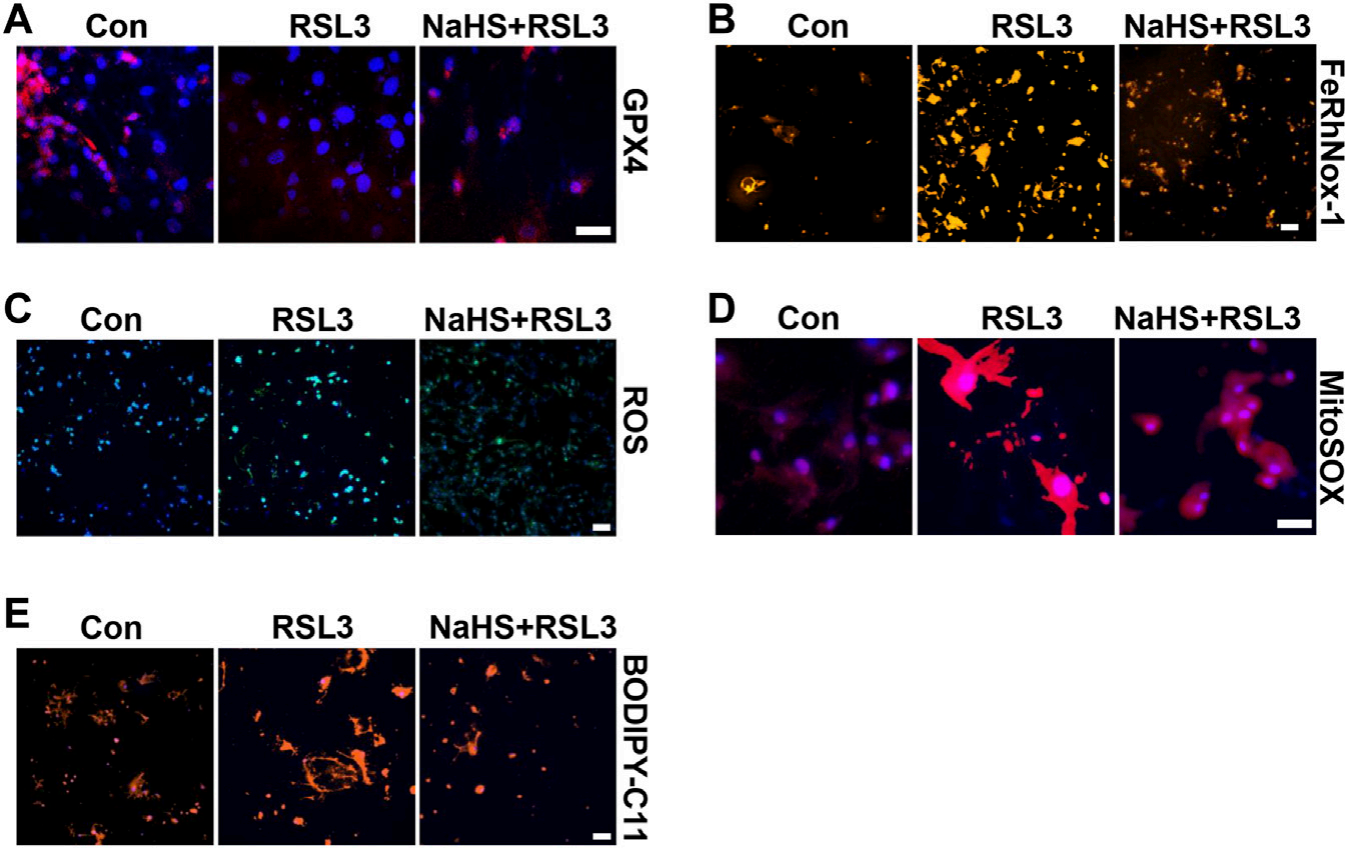
CSE/H_2_S rescue RLS3-induced ferroptosis under HHP conditions. Under 200 mmHg conditions, HASMCs were pretreated with NaHS 24 h and followed by RLS3 treatment for another 6 h. Immunofluorescent staining of GPX4 **(A)**. HASMCs were incubated with the FeRhNOX-1 probe for measuring iron **(B)**. HASMCs were labeled with the ROS probe to detect cellular ROS **(C)**, MitoSOX Red to detect mitochondrial ROS **(D)**, and BODIPY-C11 to detect lipid peroxidation **(E)** by immunofluorescent staining. All scale bar = 50 μM.

To confirm the effect of CSE/H_2_S on ferroptosis, we first used RLS3 to induce ferroptosis. In line with the above findings, NaHS administration also rescued the RLS3-induced GPX4 expression (Figure 6A and Supplementary Figure S4A), iron accumulation (Figure 6B and Supplementary Figure S4B), ROS production (Figure 6C and Supplementary Figure S4C), mitochondrial ROS production (Figure 6D and Supplementary Figure S4D), and lipid oxidation (Figure 6E and Supplementary Figure S4E). In summary, our result indicated that CSE/H_2_S contributes to the regulation of ferroptosis under HHP conditions.

## Discussion

Mechanical forces play an important role in vasculature and circulation, such as rapid regulation of vascular wall elasticity, administration of vascular remodeling, and the modulation of VSMC and endothelial function (Qiu et al., 2014). Our previous study identifies a novel cellular taxonomy of VSMCs under hydrostatic pressure by single-cell RNA sequencing. HHP derived VSMCs into inflammatory and endothelial-function inhibitory VSMCs, resulting in VSMC dysfunction (cytokine secretion and angiogenesis inhibition) (Chen Z. et al., 2021). However, the underlying mechanism is unclear. In the current study, we first demonstrated that HHP induced VSMC ferroptosis evidence as iron accumulation, ROS generation, and lipid peroxidation. Furthermore, inhibiting ferroptosis by Fer-1 alleviated, promoting ferroptosis by RLS3 increased inflammatory and endothelial function inhibitory VSMC phenotypes. For the mechanism, we demonstrated that HHP downregulated CSE/H_2_S and then lowered GSH generation, resulting in ferroptosis. Our findings highlight that ferroptosis is a novel pathophysiological regulatory mechanism for VSMC function and as a new druggable target for therapeutic hypertension concomitant vascular diseases.

Hypertension is tightly associated with oxidation stress (McMaster et al., 2015; Touyz et al., 2018). An imbalance between the oxidant and antioxidant system leads to ROS production exaggeration in the arterial media from hypertension patients with hypertension and experimental animal models of hypertension (Touyz and Schiffrin, 2001). Overproduced ROS promoted VSMC proliferation, induced VSMC migration, and switched to the differentiated phenotype (Badran et al., 2020). Dedifferentiated VSMCs synthesize and secrete a repertoire of growth factors and inflammatory cytokines which is a key initiating factor for vascular remodeling. ROS promotes MMP2, MMP9, and collagen expressions and disturbs the extracellular matrix (Rajagopalan et al., 1996; Branchetti et al., 2013). Additionally, oxidation stress induces calcification, stiffness, and aging leading to VSMC dysfunction (Takaishi et al., 2021). ROS overproduction, a center of oxidation stress, is a main characteristic of ferroptosis. Our study first identified GPX4, a key enzyme of ferroptosis, which was dramatically downregulated in arterial media of SHR. *In vitro,* VSMCs exhibited intracellular and mitochondrial ROS overproduction, lipid peroxidation, and iron accumulation under high hydrostatic stress. Ferroptosis upregulation under high hydrostatic stress is a novel pathophysiological process in VSMC dysfunction.

Compelling evidence has indicated that ferroptosis is an essential regulator of inflammation. GPX4 deficiency blocked T-cell survival and B-cell development (Matsushita et al., 2015; Muri et al., 2019). The ferroptosis inducer erastin promoted human peripheral blood mononuclear cells differentiating into B cells and natural killer cells (Tang et al., 2021). Ferroptotic death cells recruited macrophages by secreting CCL2 and CCL7 (Wang et al., 2021a). Ferroptosis inhibitors exhibited the anti-inflammation effect by suppressing TNF-α, IL-6, and IL-1β releasing (Tsurusaki et al., 2019). Consistent with the previous study, Fer-1 treatment attenuated, while RLS3 treatment augmented the HHP-stimulated CXCL2 (one marker gene of inflammatory VSMCs) protein level. In addition, high hydrostatic pressure also induced *AKR1C2* (one marker gene of endothelial function inhibitory VSMCs) which was lowered by Fer-1 but enhanced by RLS3. Studies indicated ferroptosis led to endothelial dysfunction which is a critical pathogenetic factor of hypertension. Iron accumulation resulted in endothelial damage (Vinchi et al., 2020). Fer-1 administration attenuated ox-LDL-induced inflammation and endotheliocyte death, while erastin induced ROS production and ferroptotic death of HUVECs (Xiao et al., 2019; Bai et al., 2020). Combined with our results, ferroptosis may be a potential therapeutic target to hypertension *via* modulating endothelial function.

One important characteristic of ferroptosis is GSH depletion. GSH, an intracellular antioxidant, is the synthesis from the homocysteine/methionine cycle (Rodrigues and Percival, 2019). GSH precursor l-cysteine is also a main source of hydrogen sulfide. Growing evidence demonstrated that H_2_S enhances GSH production to attenuate oxidative stress. In a neurocyte, mitochondrial H_2_S production increases the GSH level and promotes its redistribution to the mitochondria to protect the neurocyte from oxidative stress (Kimura et al., 2010). In a myotube, H_2_S promotes GSH synthesis to improve impaired glucose homeostasis (Parsanathan and Jain, 2018). H_2_S donor NaHS administration enhances GSH production to decrease oxidative stress and delay cell senescence (Yang et al., 2013). Consistently, our study showed that exogenous (NaHS administration) H_2_S production significantly rescued GSH reduction in response to HHP. Thus, H_2_S downregulation–mediated GSH reduction may be a novel mechanism of ferroptosis under HHP.

Recently, studies showed the protective role of H_2_S is associated with ferroptosis inhibition. Wang and Chen et al. identified H_2_S restrains ferroptosis *via* inhibiting ALOX12 acetylation and regulating the xCT (the functional submit of the Xc^−^ system) stability (Chen S. et al., 2021; Wang et al., 2021c). H_2_S donor GYY4137 treatment alleviates ferroptosis to attenuate acute lung injury (Li et al., 2021). Inhibition of H_2_S production *via* the CBS inhibitor CH004 supplement aggravates ferroptosis in hepatocellular carcinoma (Wang et al., 2018). These studies suggested H_2_S could restrain ferroptosis to exert a protective effect. However, experimental evidence in the cardiovascular system is still missing. Our present study illustrated that H_2_S donor NaHS administration significantly increased the GPX4 expression, downregulated ROS production, and lipid peroxidation, thus reversing high hydrostatic-induced ferroptosis. In addition, NaHS rescued RLS3-induced ferroptosis. Overall, H_2_S inhibits ferroptosis to attenuate HHP-induced VSMC dysfunction.

Taken together, the present study highlights the essential regulator role of ferroptosis in the HHP-triggered VSMC novel phenotype switch. The novel pathophysiological process also accounts for HHP, a direct biomechanical force, triggers VSMC ferroptosis, then drives vascular inflammation and limitation vascular relaxation, and contributes to vascular remodeling and aging even if calcified, thus exacerbating concomitant vascular damages and diseases (such as atherosclerosis). More interestingly, our study also indicates a new pathway about the ROS-GSH-iron-ferroptosis signal cascade in CSE/H_2_S cardiovascular protection.

## Data Availability

The original contributions presented in the study are included in the article/Supplementary Material; further inquiries can be directed to the corresponding authors.
