# Supplementary material for: Ferroptosis due to Cystathionine γ Lyase/Hydrogen Sulfide Downregulation Under High Hydrostatic Pressure Exacerbates VSMC Dysfunction
Source: Front Cell Dev Biol. 2022 Feb 3;10:829316. doi: 10.3389/fcell.2022.829316 (PMC8850391; doi:10.3389/fcell.2022.829316)
Supplement: Supplementary file 1 [file DataSheet1.docx]

Supplementary Material

**Table S1.** **List of oliGonucleotide primer pairs used in qRT-PCR and analysis.**

| TarGet Gene | Forward primer (5'-3') | Reverse primer(5'-3') |
| --- | --- | --- |
| COX-2 (H) | CTGGCGCTCAGCCATACAG | CGCACTTATACTGGTCAAATCCC |
| NOX-1 (H) | TTGTTTGGTTAGGGCTGAATGT | GCCAATGTTGACCCAAGGATTT |
| GPX4 (H) | GAGGCAAGACCGAAGTAAACTAC | CCGAACTGGTTACACGGGAA |
| TFRC (H) | ACCATTGTCATATACCCGGTTCA | CAATAGCCCAAGTAGCCAATCAT |
| SLC7A11 (H) | TCTCCAAAGGAGGTTACCTGC | AGACTCCCCTCAGTAAAGTGAC |
| ACSL4 (H) | CATCCCTGGAGCAGATACTCT | TCACTTAGGATTTCCCTGGTCC |
| CSE (H) | AAAGACGCCTCCTCACAAGG | AAGGCAATTCCTAGTGGGATTTC |
| GAPDH (H) | CTGGGCTACACTGAGCACC | AAGTGGTCGTTGAGGGCAATG |

H: human


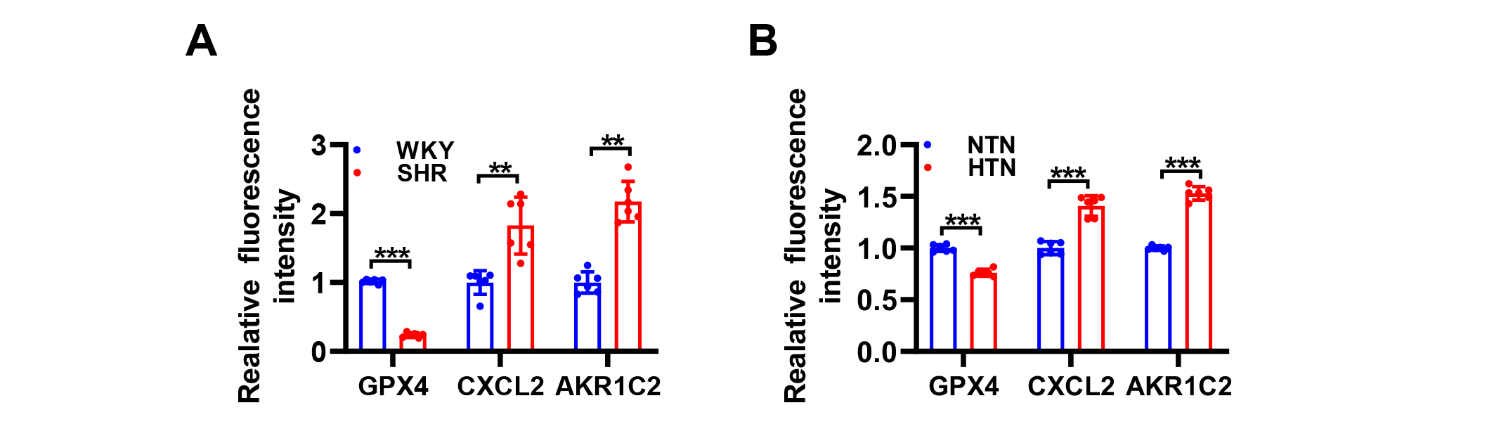


**Figure S1. GPX4 expression and VSMCs specific phenotype are increase in the aortal media of SHR and hypertensive patients.** The statistical graph of GPX4, CXCL2 and AKR1C2 immunofluorescent in the arterial media of SHR **(A)**. The statistical graph of GPX4, CXCL2 and AKR1C2 immunofluorescent in human normotension (NTN) and hypertension (HTN) internal mammary arteries **(B)**. ***P*<0.01, ****P*<0.001.


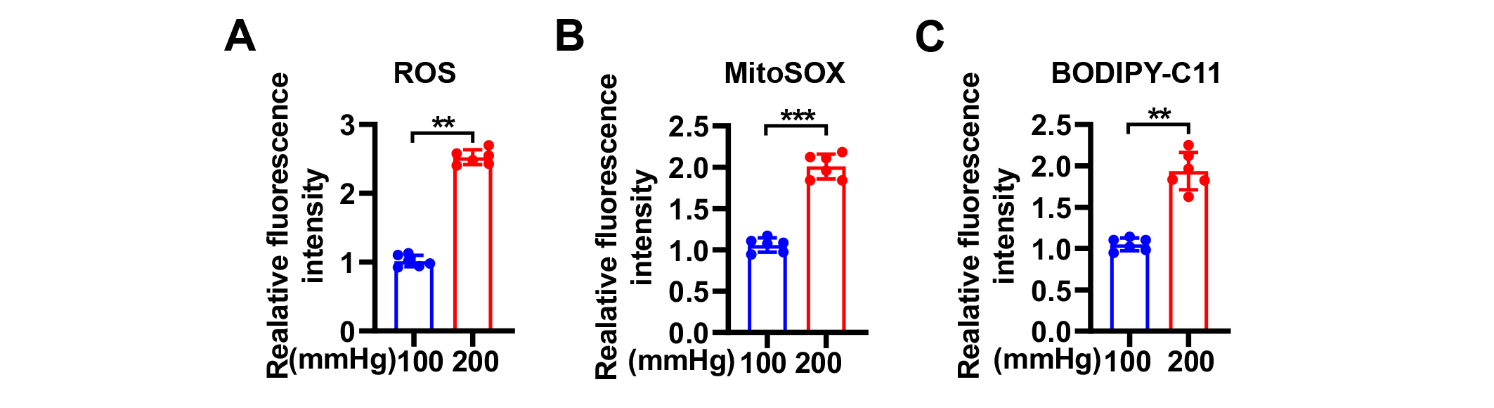


**Figure S2. HHP induced oxidative stress and lipid peroxidation.** The statistical graph of ROS **(A)**, MitoSOX **(B)** and lipid peroxidation **(C)** of HASMCs under HHP condition. ***P*<0.01, ****P*<0.001.


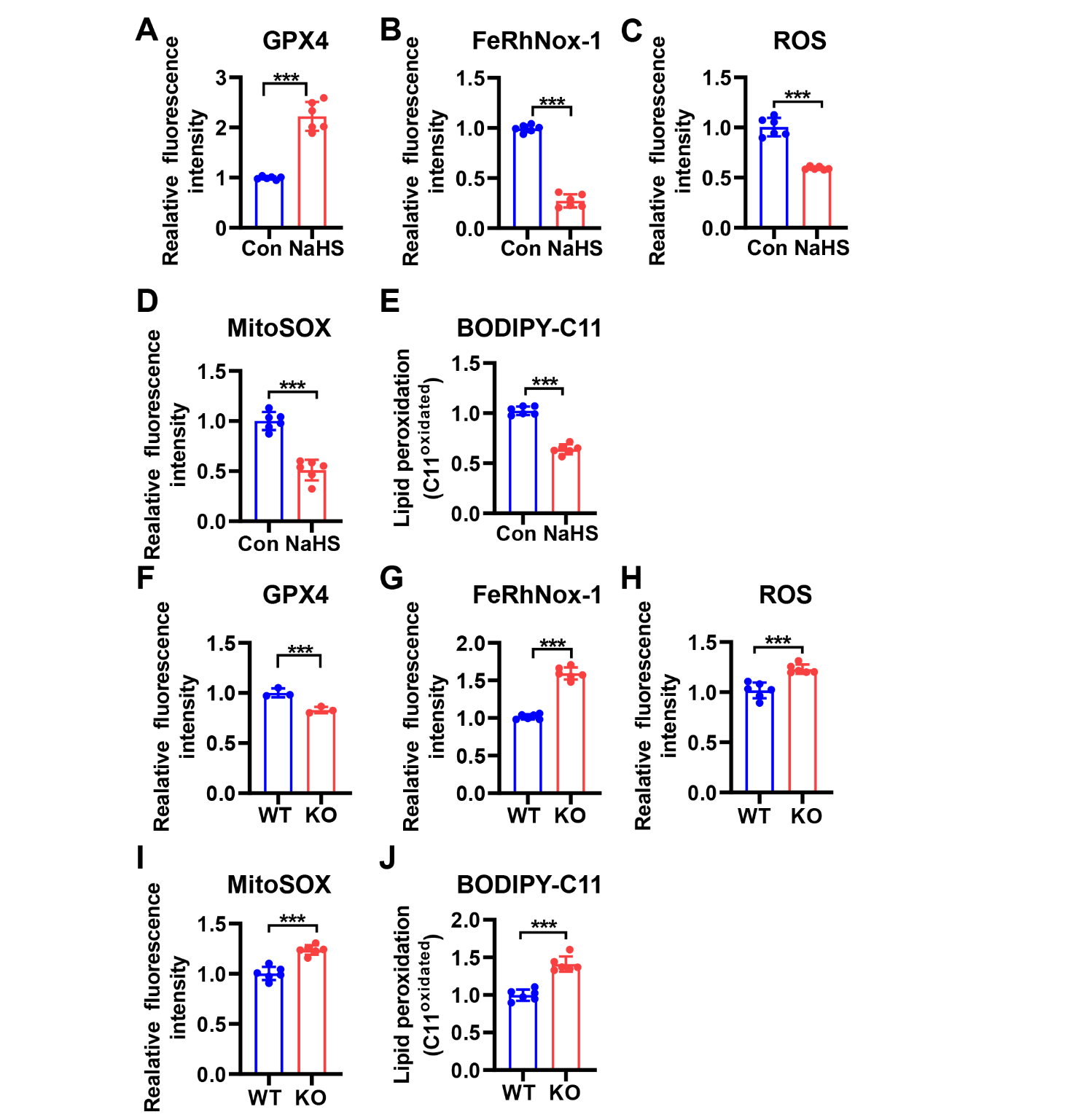


**Figure S3. CSE/H_2_S rescued HHP-induced ferroptosis.** The effect of exogenous CSE/H_2_S increase (NaHS administration) on ferroptosis under HHP condition. The statistical graph of GPX4 **(A)**, Fe^2+^ **(B)**, ROS **(C)**, mitochondrial ROS **(D)** and BODIPY-C11 **(E)** immunofluorescent. The effect of endogenous CSE/H_2_S decrease (CSE knockout) in ferroptosis under HHP condition. The statistical graph of GPX4 **(F)**, Fe^2+^ **(G)**, ROS **(H)**, mitochondrial ROS **(I)** and BODIPY-C11 **(J)** immunofluorescent. ****P*<0.001.


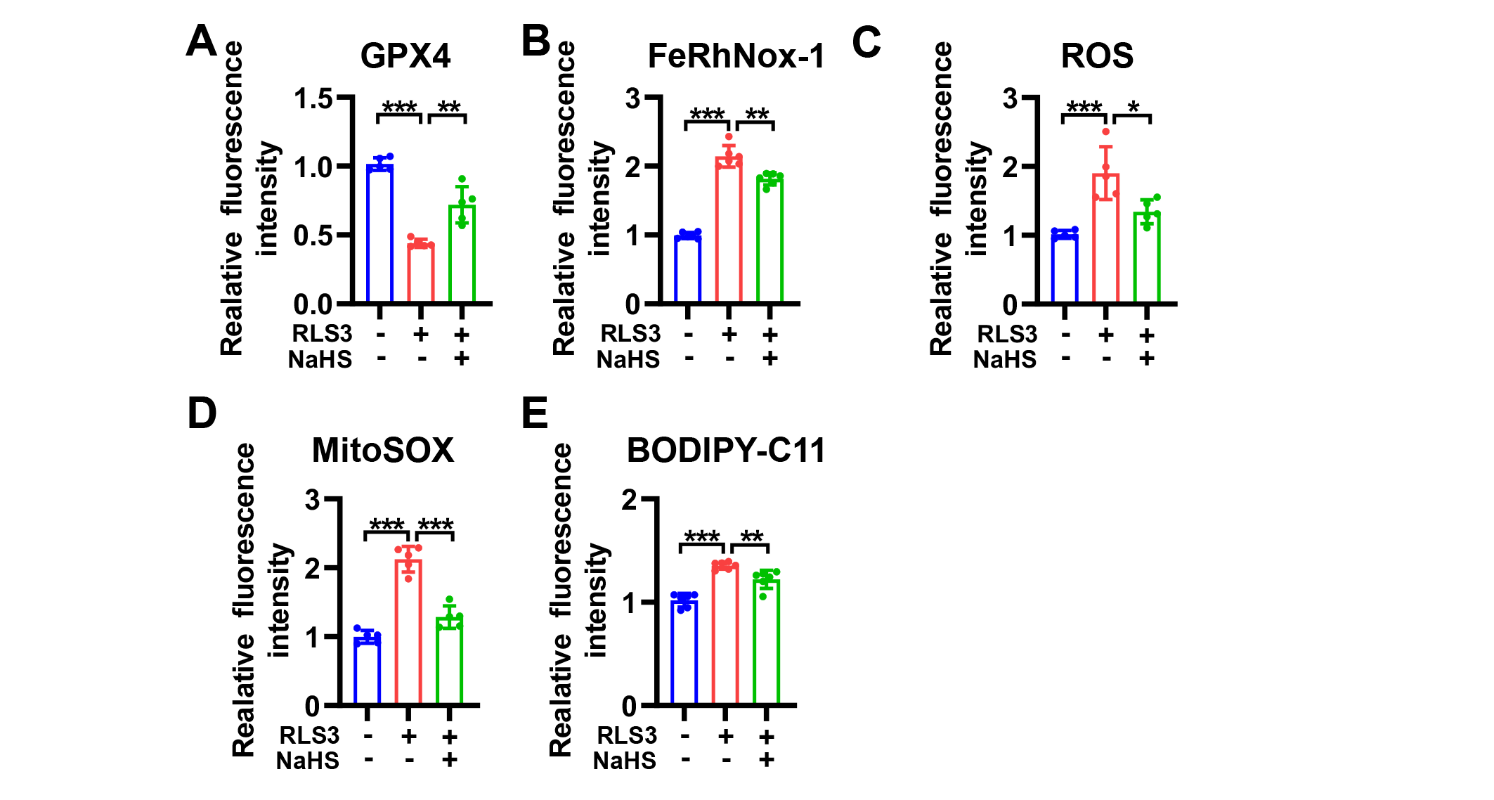


**Figure S4. NaHS rescued RLS3-induced ferroptosis.** The statistical graph of GPX4 The statistical graph of Figure 6A-E **(A-E)**. **P*<0.05, ***P*<0.01, ****P*<0.001.
